# Identification of candidate genes of intellectual disability by single-gene deletions/amplifications mapping using chromosomal microarray analysis

**DOI:** 10.1192/j.eurpsy.2022.969

**Published:** 2022-09-01

**Authors:** A. Kashevarova, M. Lopatkina, E. Belyaeva, D. Fedotov, G. Drozdov, L. Nazarenko, I. Lebedev

**Affiliations:** Research Institute of Medical Genetics, Tomsk National Research Medical Center, Russian Academy of Sciences, Laboratory Of Ontogenetics, Tomsk, Russian Federation

**Keywords:** intellectual disability, Single-gene deletion, Single-gene amplification

## Abstract

**Introduction:**

Disease-causing deletions/amplifications may include a single gene, several exons or single/part of exon, contributing to detection of novel pathogenic genes. The localization of single-gene deletion/amplification within the gene can affect its clinical manifestation.

**Objectives:**

Improvement of diagnosis of intellectual disability.

**Methods:**

aCGH with 60K Agilent microarrays, qPCR.

**Results:**

Among 1099 patients with intellectual disability potentially pathogenic single-gene deletions/amplifications were detected in 51 individuals (5%). qPCR was used to verify aberrations in 21 patients (41%). Ten mutations were of maternal origin, four - paternal, two - *de novo*, another two were confirmed without analysis of parents, and three could not be confirmed. Single-gene aberrations involving the *AGBL4* (exon 2), *ASMT* (exon 9), *CYP2C18* (whole gene)*, DDX10* (promoter, exons 1-13)*, GYPA* (whole gene), *LIG4* (exon 1)*, LSAMP* (intron 1)*, PSD3* (promoter, exons 1-11), *SNTB1* (intron 1), *SPOCK3* (exons 6-12)*, STAG2* (exons 7-34)*, SYT10* (promoter, exons 1-2)*, TCAF2* (exon 8)*, TMPRSS15* (promoter, exons 1-12), and *ZDHHC7* (promoter, exons 1-4) genes were described by us for the first time. Deletion or amplification of several exons within a gene can affect transcription as point mutation does, while the copy number change of a whole gene can lead to an abnormal amount of the protein.

**Conclusions:**

Fifteen novel genes potentially responsible for mental health were identified. In most of them aberrations were partial deletions/duplications. Most of abnormalities were inherited from healthy parents indicating the possible presence of a point mutation on the second allele or some modifying factors. This study was supported by the Russian Science Foundation, grant 21-65-00017.

**Disclosure:**

No significant relationships.

